# Psychological and quality of life outcomes associated with multikinase inhibitors versus immune checkpoint inhibitors in advanced hepatocellular carcinoma

**DOI:** 10.1038/s41598-026-39864-y

**Published:** 2026-02-12

**Authors:** Maher Hendi, Jie-Min Lv, Mohamad Hndi, Bin Zhang, Xue-Qin Chen, Ying-Ying Chen, Yu Pan, Xiu-Jun Cai

**Affiliations:** 1 Department of Surgery, Zhejiang University School of Medicine, Sir Run Run Shaw Hospital, No. 3 Qingchun Road, Hangzhou, 310016 Zhejiang Province China; 2 Zhejiang University School of Medicine, Hangzhou, 310058 Zhejiang Province China; 3 Department of Gastroenterology, Zhejiang University School of Medicine, Sir Run Run Shaw Hospital, Hangzhou, 310016 Zhejiang Province China; 4 Department of Endoscopy Centre, Zhejiang University School of Medicine, Sir Run Run Shaw Hospital, Hangzhou, 310016 Zhejiang Province China

**Keywords:** Hepatocellular carcinoma, Multikinase inhibitors, Immune checkpoint inhibitors, Anxiety, Depression, Cancer, Oncology

## Abstract

To compare the effects of multikinase inhibitors (sorafenib/lenvatinib) and immune checkpoint inhibitors (PD-1/PD-L1) on anxiety, depression, and quality of life (QoL) in patients with advanced hepatocellular carcinoma (HCC) and to analyse their correlations with clinical indicators. This retrospective cohort study included 304 patients with advanced HCC (BCLC stage B/C) who received first-line monotherapy between 2018 and 2023. Propensity score matching (1:1) was used to categorize patients into two groups: those treated with multikinase inhibitors (*n* = 152) and those treated with PD-1/PD-L1 inhibitors (*n* = 152). Anxiety and depression (Hospital Anxiety and Depression Scale (HADS)), and QoL (EORTC QLQ-C30) were assessed at baseline and during follow-up (every 3 months). Correlations between the treatment duration, survival outcomes, and adverse events (AEs) were analysed. The PD-1/PD-L1 group presented significant reductions in anxiety (HADS-A: mean difference [MD] = − 2.4) and depression (HADS-D: MD = − 2.3) at 6 months (both *p < 0.001), with lower rates of clinically significant anxiety (28.3% vs. 42.1%) and depression (24.3% vs. 38.8%; *p* < 0.05). QoL improved markedly (6-month MD = + 10.3, *p* < 0.001), particularly when the treatment was administered as the first-line therapy (MD = + 14.2 vs. second-line MD = + 3.8; interaction *p* < 0.001). PD-1/PD-L1 inhibitors were associated with longer treatment durations (median 9.5 vs. 5.8 months, *p* < 0.001) and superior overall survival (median 18.2 vs. 12.5 months; HR = 0.62, *p* = 0.002). Fatigue (grade ≥ 2) independently predicted depression (OR = 1.82, *p* = 0.002), whereas immune-related AEs were correlated with a reduced QoL (ρ=−0.22, *p* = 0.004). Compared with multikinase inhibitors, PD-1/PD-L1 inhibitors significantly improve the psychological outcomes, QoL, and survival of patients with advanced HCC, especially when administered as first-line therapies. Fatigue is a critical modifiable risk factor for depression. These findings support prioritizing immunotherapy when treating atients with advanced hepatocellular carcinoma.

**Supplementary Information:**

The online version contains supplementary material available at 10.1038/s41598-026-39864-y.

## Introduction

Hepatocellular carcinoma (HCC) remains a significant global health challenge, ranking as the sixth most common cancer and the third leading cause of cancer-related mortality worldwide^1^. More than 90% of cases develop in the context of chronic liver disease, with hepatitis B virus, hepatitis C virus, alcohol-related liver disease, and nonalcoholic fatty liver disease representing major aetiological factors^2,3^. Despite advancements in surveillance programs, approximately half of patients are diagnosed with HCC at advanced stages, where curative interventions such as resection or transplantation are no longer feasible^4^. The Barcelona Clinic Liver Cancer (BCLC) staging system recommends systemic therapy as the standard of care for patients with advanced HCC, with multikinase inhibitors such as sorafenib and lenvatinib, as well as immune checkpoint inhibitors targeting PD-1 or PD-L1, forming a therapeutic foundation^5,6^. However, geographical disparities in treatment access persist, and five-year survival rates remain less than 20% in most regions, underscoring the need for optimized therapeutic strategies^7^.

The introduction of sorafenib marked a pivotal advancement in the management of advanced HCC, and it resulted in a median overall survival benefit of 10.7 months compared with the placebo^8^. Subsequent studies established lenvatinib as noninferior to sorafenib, with improved radiological response rates but comparable survival outcomes^9^. The emergence of PD-1 and PD-L1 inhibitors, including nivolumab and pembrolizumab, further expanded the treatment options, resulting in durable responses in a subset of patients during early phase trials^10^. Landmark studies such as IMbrave150 later documented superior survival outcomes of patients treated with combination therapy involving atezolizumab and bevacizumab, achieving a median overall survival of 19.2 months compared with that of patients receiving sorafenib, thereby establishing immunotherapy as a first-line standard treatment^11^.

Despite these advancements, therapeutic regimens exhibit distinct toxicity profiles: multikinase inhibitors are frequently associated with hypertension, hand–foot syndrome, and fatigue^10^, whereas immune checkpoint inhibitors carry risks of immune-related adverse events such as hepatitis and pneumonitis^12^. These divergent safety profiles highlight the importance of personalized treatment approaches that account for patients’ comorbidities and psychosocial resilience.

Psychosocial distress affects patients with advanced HCC and is often exacerbated by disease-related symptoms, treatment toxicity, and prognostic uncertainty^13^. The evidence indicates that psychological morbidity correlates with reduced treatment adherence and poor survival outcomes^14^. The effects of different systemic therapies on these psychological parameters require specific attention in contemporary HCC management. Multikinase inhibitors (MKIs) have been associated with significant toxicity, including hand–foot skin reactions, diarrhoea, and fatigue, in up to 80% of patients^15^. These adverse events have been linked to decreased functioning and elevated anxiety in several cohort studies^16,17^. Second-generation MKIs maintain similar toxicity patterns that can interfere with psychosocial well-being^10^.

In contrast, the psychological effects of immune checkpoint inhibitors (ICIs) on patients with HCC remains underexplored. Evidence obtained from patients with other solid tumours suggests that ICIs may have more favourable psychological profiles because of distinct toxicity patterns and extended response durations^18^. Comparative studies of patients with lung cancer and melanoma described a better preservation of quality of life with immunotherapy than with conventional treatments^19,20^. However, immune-related adverse events present unique challenges that require dedicated investigations in HCC populations^21^.

Despite these potential differences, direct comparative data on psychosocial outcomes in patients with HCC treated with these different classes of therapeutic agents are notably absent from the literature.

Recent pivotal trials establishing the use of ICIs for HCC management have not comprehensively assessed anxiety, depression, or detailed quality of life domains^22^. This research gap is particularly concerning given that psychological outcomes may substantially influence treatment selection and adherence in real-world settings.

This study addresses a critical unmet need by providing the first direct comparison of the effects of PD-1/PD-L1 inhibitors and multikinase inhibitors on the psychosocial outcomes in patients with advanced HCC. Given their distinct mechanisms of action and toxicity profiles, we aimed to determine whether differences exist in patient-reported anxiety, depression, and quality of life using validated psychometric instruments while controlling for relevant clinical factors. Additionally, we explored whether the treatment sequence (first-line versus second-line therapy) modifies these relationships, as responsiveness may vary by prior treatment exposure. These findings may inform a more holistic approach to treatment selection that considers not only efficacy endpoints but also the effects on psychological well-being, providing clinicians with comprehensive data to guide patient-centred decision-making in contemporary HCC management.

## Methods

### Study design

This retrospective cohort study analysed patients with advanced HCC who were treated at Sir Run Run Shaw Hospital of Zhejiang University School of Medicine between January 2018 and December 2023. The study protocol was approved by the Institutional Review Board of the Zhejiang University School of Medicine Sir Run Run Shaw Hospital (Approval No. 2024 − 114 and Acceptance No. 2024-2613-01), and informed consent requirements were waived due to the retrospective design. The data were extracted from institutional electronic medical records (EMRs) and a structured psychosocial assessment database. Data collection focused on baseline psychological evaluations (anxiety, depression, and quality of life [QoL]), longitudinal psychosocial assessments during clinical follow-up, treatment duration, and adverse event documentation. Study procedures adhered to the Declaration of Helsinki, and patient confidentiality was maintained through anonymized data handling.

### Participant selection

Patients with histologically or radiologically confirmed advanced HCC were screened for eligibility. The inclusion criteria were as follows: (1) BCLC stage B or C disease, consistent with the guidelines for systemic therapy initiation for unresectable HCC^23^; (2) initiation of first-line monotherapy with either multikinase inhibitors (sorafenib/lenvatinib) or immune checkpoint inhibitors (PD-1/PD-L1); (3) survival ≥ 6 months after treatment initiation to ensure adequate follow-up for longitudinal psychosocial assessments; and (4) the completion of ≥ 2 structured psychosocial evaluations during the study period. The exclusion criteria were as follows: (1) preexisting psychiatric disorders (e.g., major depressive disorder or generalized anxiety disorder) documented in the patients’ medical records, as these conditions may confound psychological outcome measures; (2) cognitive impairment (e.g., dementia or delirium) affecting the validity of self-reported psychosocial assessments; (3) the receipt of combination therapies (e.g., tyrosine kinase inhibitors with immunotherapy) to isolate the effects of monotherapy regimens; and (4) incomplete baseline or follow-up psychosocial data. The participants were categorized into two exposure groups based on their first-line systemic therapeutic regimen, as documented in the electronic medical records: Group 1 received multikinase inhibitor monotherapy (sorafenib or lenvatinib), and Group 2 received immune checkpoint inhibitor monotherapy (PD-1/PD-L1 inhibitors). Treatment selection was guided by the physician’s discretion, patient comorbidities, and institutional protocols during the study period (2018–2023). Propensity score matching (PSM) was applied using a 1:1 nearest-neighbour algorithm without replacement to address potential confounding biases inherent to retrospective observational data. The covariates for matching included age (± 5 years), Child‒Pugh class (A vs. B/C), and baseline tumour burden (unilobar vs. bilobar involvement), ensuring balanced baseline characteristics between groups. All analyses adhered to an intention-to-treat framework; patients analysed according to their initial treatment assignment, regardless of subsequent therapeutic modifications.

### Data collection

Clinical, psychosocial, and treatment-related data were retrospectively extracted from institutional EMRs and a structured psychosocial assessment database. Baseline clinical and demographic data included demographic variables (age, sex, and marital status) and clinical characteristics (Child‒Pugh class, tumour burden [unilobar vs. bilobar], ECOG performance status, and aetiology of HCC [HBV, HCV, or other]) and were retrieved from hepatology consultations and imaging reports. Laboratory parameters (albumin and total bilirubin levels) and staging criteria (BCLC stage) were extracted from pretreatment diagnostic evaluations. Baseline psychological evaluations included pretreatment anxiety and depression scores measured by the Hospital Anxiety and Depression Scale (HADS) and QoL assessed using the EORTC QLQ-C30 Global Health Status subscale (validated Chinese version)^24^.

Longitudinal psychosocial assessments were synchronized with radiologic re-evaluation visits during clinical follow-up (every 3 months) and at treatment discontinuation or patient death. Adverse events (AEs) were systematically documented according to the Common Terminology Criteria for Adverse Events (CTCAE v5.0)^25^, with the severity graded as mild (Grade 1), moderate (Grade 2), severe (Grade 3), or life-threatening (Grade 4). Key AEs of interest included fatigue, hypertension, dermatologic reactions (e.g., hand‒foot syndrome), and immune-related adverse events (irAEs; e.g., hepatitis and pneumonitis).

The HADS and EORTC QLQ-C30 assessments were independently reviewed by two trained psychologists who were blinded to the treatment allocation to ensure data accuracy. Discrepancies in scoring (e.g., ambiguous responses) were resolved through adjudication by a third senior investigator. Continuous HADS scores (ranging from 021 per subscale) were analysed as follows: continuous variables and using validated cut-off thresholds (≥ 8 for clinically significant anxiety/depression)^26^.

EORTC QLQ-C30 Global Health Status scores were transformed to a 0–100 point scale, with higher values indicating better QoL^27^. Assessment compliance was high, with 92% of the scheduled evaluations completed across all time points. Missing data (< 10%) were due primarily to missed appointments (4%), a deteriorating performance status (3%), or administrative errors (3%). The pattern of missingness was evaluated and determined to be missing at random (MAR). Treatment duration was calculated from initiation to discontinuation (due to progression, toxicity, or death). The radiological tumour burden (unilobar vs. bilobar involvement) and Child‒Pugh class were extracted from imaging reports and hepatology consultations. Treatment Line Definitions: First-line therapy was defined as the initial systemic treatment for advanced HCC, with no prior exposure to systemic agents (e.g., tyrosine kinase inhibitors or immunotherapy), consistent with the Barcelona Clinic Liver Cancer (BCLC) guidelines for unresectable HCC^23^.

Second-line therapy was defined as systemic treatment initiated after documented radiological progression (according to RECIST 1.1^28^) or intolerance to first-line therapy (e.g., sorafenib/lenvatinib followed by PD-1/PD-L1 inhibitors, or vice versa).

### Statistical analysis

Statistical analyses were performed using R software (version 4.3.1, https://www.r-project.org ) (R Foundation for Statistical Computing, Vienna, Austria) and STATA version 17.0 (StataCorp, College Station, TX, USA), with a two-sided α level of 0.05 indicating significance.

Longitudinal changes in anxiety/depression (HADS scores) and quality of life (EORTC QLQ-C30 Global Health Status) were analysed using linear mixed-effects models to account for repeated measures and individual variability over time. These models included fixed effects for the treatment group, time, treatment-by-time interaction, age, Child‒Pugh class, and baseline tumour burden, with random intercepts and slopes for individual patients to account for between-subject heterogeneity. Model assumptions were verified using residual diagnostics. Group comparisons between patients treated with multikinase inhibitors (sorafenib/lenvatinib) and immune checkpoint inhibitors (PD-1/PD-L1) were conducted after propensity score matching (PSM) with 1:1 nearest neighbour matching without replacement (calliper width: 0.2 standard deviations of the logit of the propensity score) and after adjusting for age, the Child‒Pugh class, and baseline tumour burden. The balance of covariates before and after matching was assessed using standardized mean differences, with values < 0.1 indicating an adequate balance. Treatment effects were estimated from the matched cohort while accounting for the matched-pair design (Supplementary Table S1). Continuous outcomes are reported as the mean differences with 95% confidence intervals (CIs), whereas categorical outcomes (e.g., clinically significant anxiety/depression defined by a HADS score ≥ 8) were analysed using logistic regression. Correlations between adverse events and psychological outcomes were assessed using Spearman’s rank correlation coefficients for nonnormally distributed data. Subgroup analyses stratified by treatment line (first-line vs. second-line) employed interaction terms within regression models to evaluate the heterogeneity of the effects. Sensitivity analyses included multiple imputation by chained equations (MICEs) for missing psychosocial data (< 10% missingness) and complete-case comparisons to validate robustness. The survival duration was analysed using Kaplan‒Meier curves with log-rank tests and was censored at the last follow-up or non-HCC-related death. All analyses adhered to intention-to-treat principles, and model assumptions (e.g., normality and homoscedasticity) were verified using residual diagnostics.

## Results

### Comparison of the baseline characteristics

After PSM, 152 patients were included in each treatment group (sorafenib/lenvatinib vs. PD-1/PD-L1 inhibitors), with balanced baseline characteristics. Key clinical and psychosocial variables were incorporated to address potential confounding factors (Table 1). Prior to matching, the PD-1/PD-L1 group had a higher proportion of Child‒Pugh class A patients (78% vs. 65%, *p* = 0.012), younger patients (mean 58.2 vs. 62.5 years, *p* = 0.003), and patients with a better baseline Eastern Cooperative Oncology Group Performance Status (ECOG PS 0–1: 88% vs. 72%, *p* < 0.001). Following PSM, standardized differences for all covariates (age, Child‒Pugh class, tumour burden, ECOG PS, aetiology, and laboratory parameters) were < 10%, indicating a robust balance. Both groups had comparable baseline anxiety/depression scores (HADS total score: 12.3 vs. 12.1; *p* = 0.72) and quality of life (EORTC QLQ-C30 Global Health Status: 52.4 vs. 53.8; *p* = 0.41) across all follow-up visits.

**Table 1:** Baseline characteristics before and after propensity score matching.

Characteristic	Before Matching	After Matching		Before Matching	After Matching		
	Sorafenib/Lenvatinib (*n* = 180)	PD-1/PD-L1 (*n* = 170)	*p*-value	Sorafenib/Lenvatinib (*n* = 152)	PD-1/PD-L1 (*n* = 152)	Std. Diff (%)	*p*-value
Age, years	62.5 ± 9.8	58.2 ± 10.3	0.003	59.1 ± 9.5	58.7 ± 9.9	4.1	0.68
Male sex	128 (71.1%)	122 (71.8%)	0.89	109 (71.7%)	108 (71.1%)	1.3	0.91
Child-Pugh class			0.012			3.2	0.82
- A	117 (65.0%)	133 (78.2%)		116 (76.3%)	118 (77.6%)		
- B/C	63 (35.0%)	37 (21.8%)		36 (23.7%)	34 (22.4%)		
Tumor burden			0.18			6.8	0.54
- Unilobar	92 (51.1%)	98 (57.6%)		83 (54.6%)	85 (55.9%)		
- Bilobar	88 (48.9%)	72 (42.4%)		69 (45.4%)	67 (44.1%)		
ECOG PS			< 0.001			7.2	0.63
− 0–1	130 (72.2%)	150 (88.2%)		128 (84.2%)	130 (85.5%)		
- ≥2	50 (27.8%)	20 (11.8%)		24 (15.8%)	22 (14.5%)		
Etiology			0.22			8.5	0.41
- HBV	112 (62.2%)	105 (61.8%)		95 (62.5%)	97 (63.8%)		
- HCV	45 (25.0%)	38 (22.4%)		40 (26.3%)	36 (23.7%)		
- Other	23 (12.8%)	27 (15.9%)		17 (11.2%)	19 (12.5%)		
Extrahepatic metastasis	68 (37.8%)	55 (32.4%)	0.29	58 (38.2%)	56 (36.8%)	3.0	0.81
Albumin, g/dL	3.4 ± 0.6	3.6 ± 0.5	0.006	3.5 ± 0.5	3.5 ± 0.6	5.8	0.72
Total bilirubin, mg/dL	1.8 ± 0.9	1.5 ± 0.7	0.002	1.6 ± 0.8	1.6 ± 0.7	2.1	0.88
Marital status			0.45			9.3	0.56
- Married	125 (69.4%)	123 (72.4%)		108 (71.1%)	110 (72.4%)		
- Single/Divorced/Widowed	55 (30.6%)	47 (27.6%)		44 (28.9%)	42 (27.6%)		
Baseline HADS total	12.3 ± 3.5	12.1 ± 3.8	0.72	12.2 ± 3.6	12.0 ± 3.7	5.5	0.65
EORTC QLQ-C30 (GHS)	52.4 ± 14.2	53.8 ± 13.6	0.41	53.1 ± 13.9	53.5 ± 14.1	2.9	0.78

Data presented as mean ± SD or n (%). Std. Diff = Standardized difference; GHS = Global Health Status; ECOG PS = Eastern Cooperative Oncology Group Performance Status. Statistical tests: Continuous variables analyzed via independent t-test; categorical variables via chi-square or Fisher’s exact test.

### Longitudinal changes in anxiety and depression (HADS scores)

**Fig. 1:**
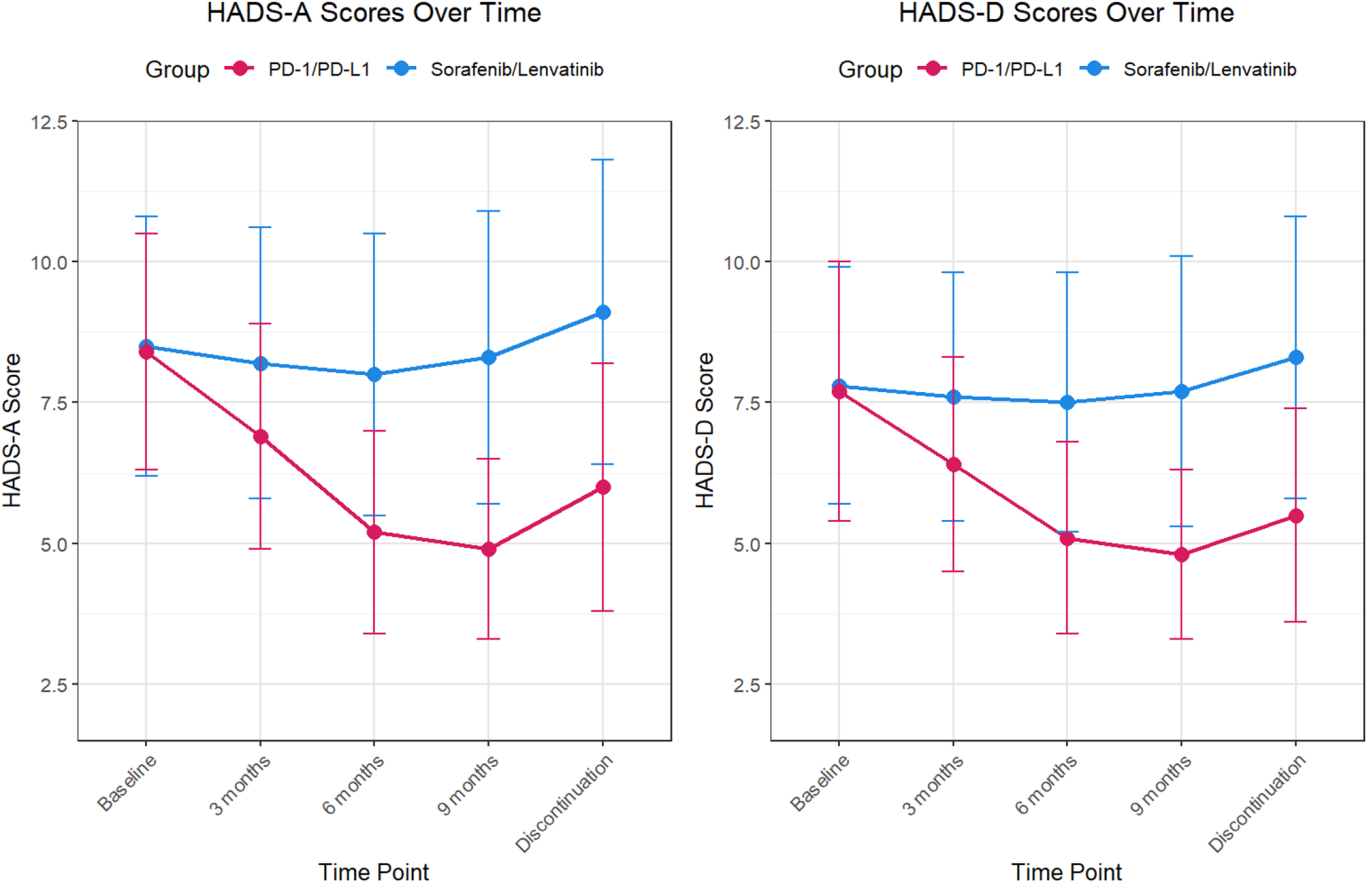
Longitudinal changes in Hospital Anxiety and Depression Scale (HADS) scores between the PD-1/PD-L1 inhibitor group and the sorafenib/lenvatinib group. The data are presented as the means ± SDs. HADS-A = Hospital Anxiety and Depression Scale–Anxiety subscale; HADS-D = Hospital Anxiety and Depression Scale–Depression subscale.

A longitudinal analysis of HADS scores revealed distinct trajectories between the two treatment groups. Patients receiving PD-1/PD-L1 inhibitors exhibited a progressive reduction in anxiety (HADS-A) and depression (HADS-D) scores over time, whereas the multikinase inhibitor group showed stable or worsening trends. At 6 months, the PD-1/PD-L1 group presented a significantly greater reduction in HADS-A scores than at baseline (mean change: −3.2 ± 1.1 vs. −0.8 ± 1.3 in the multikinase inhibitor group; adjusted mean difference [AMD]: −2.4, 95% CI: −3.1 to − 1.7, *p* < 0.001). Similarly, HADS-D scores decreased by − 2.8 ± 1.0 in the PD-1/PD-L1 group compared with − 0.5 ± 1.2 in the multikinase inhibitor group (AMD: −2.3; 95% CI: −3.0 to − 1.6; *p* < 0.001). These differences persisted at the 9-month follow-up (Fig. 1 and Supplementary Table S2).

#### Clinically significant anxiety/depression (HADS score ≥ 8)

The proportion of patients meeting the criteria for clinically significant anxiety (HADS-A score ≥ 8) or depression (HADS-D score ≥ 8) differed markedly between the treatment groups over time. At baseline, both groups had comparable rates of anxiety (sorafenib/lenvatinib: 56.6% vs. PD-1/PD-L1: 54.6%, *p* = 0.72) and depression (50.0% vs. 48.7%, *p* = 0.81). By 6 months, the PD-1/PD-L1 group exhibited significantly lower rates of anxiety (28.3% vs. 42.1%, adjusted OR = 0.55, 95% CI: 0.32–0.94, *p* = 0.028) and depression (24.3% vs. 38.8%, adjusted OR = 0.49, 95% CI: 0.28–0.85, *p* = 0.011), with sustained reductions at treatment discontinuation (Table 3). Multivariate logistic regression confirmed that PD-1/PD-L1 therapy was an independent factor protecting against clinically significant anxiety (adjusted OR = 0.54, *p* = 0.002) and depression (adjusted OR = 0.51, *p* = 0.003) across all time points (Table 2 and Supplementary Fig. S1).

**Table 2:** Prevalence of clinically significant anxiety, depression (HADS ≥ 8) and EORTC QLQ-C30 global health status.

Outcome	Timepoint	Sorafenib/Lenvatinib (*n* = 152)	PD-1/PD-L1 (*n* = 152)	Adjusted OR (95% CI)	*p*-value
Anxiety (HADS-A ≥ 8)	Baseline	86 (56.6%)	83 (54.6%)	0.92 (0.56–1.52)	0.72
	3 months	72 (47.4%)	56 (36.8%)	0.64 (0.40–1.02)	0.061
	6 months	64 (42.1%)	43 (28.3%)	0.55 (0.32–0.94)	0.028
	Discontinuation	69 (45.4%)	48 (31.6%)	0.58 (0.35–0.96)	0.033
Depression (HADS-D ≥ 8)	Baseline	76 (50.0%)	74 (48.7%)	0.95 (0.58–1.55)	0.81
	3 months	68 (44.7%)	52 (34.2%)	0.66 (0.41–1.08)	0.095
	6 months	59 (38.8%)	37 (24.3%)	0.49 (0.28–0.85)	0.011
	Discontinuation	62 (40.8%)	41 (27.0%)	0.53 (0.31–0.89)	0.017
EORTC QLQ-C30	Baseline	53.1 ± 13.9	35.5 ± 14.1	–	0.78
	3 months	50.2 ± 12.6	58.8 ± 13.2	+ 8.6 (5.3–11.9)	< 0.001
	6 months	55.2 ± 14.0	65.9 ± 12.4	+ 10.3 (7.2–13.4)	< 0.001
	Discontinuation	51.3 ± 14.5	60.1 ± 13.0	+ 8.6 (5.1–12.1)	< 0.001

Adjusted ORs derived from multivariate logistic regression models, controlling for age, Child-Pugh class, baseline tumor burden, and ECOG PS. HADS-A = Anxiety subscale; HADS-D = Depression subscale.

EORTC QLQ-C30 Global Health Status scores range from 0–100, with higher scores indicating better quality of life.

**Table 3:** Correlations between adverse events and psychological outcomes.

Adverse Event (CTCAE Grade)	HADS-A (Anxiety)	HADS-D (Depression)	EORTC QLQ-C30 (QoL)
	ρ (*p*-value)	ρ (*p*-value)	ρ (*p*-value)
Fatigue (≥ Grade 2)	0.14 (0.029)	0.28 (< 0.001)	− 0.18 (0.006)
Hypertension (≥ Grade 2)	0.19 (0.008)	0.12 (0.051)	− 0.10 (0.12)
Hand-foot syndrome (≥ Grade 3)	0.17 (0.016)	0.15 (0.021)	− 0.13 (0.034)
Immune-related AEs (≥ Grade 2)	0.09 (0.15)	0.11 (0.074)	− 0.22 (0.004)
Hepatitis (≥ Grade 3)	0.08 (0.20)	0.07 (0.25)	− 0.16 (0.011)

HADS-A/D: Hospital Anxiety and Depression Scale-Anxiety/Depression subscales (higher scores = worse symptoms). EORTC QLQ-C30: Global Health Status subscale (higher scores = better quality of life). Spearman’s rank correlation coefficients (ρ) reported with p-values.

### Quality of life trajectories (EORTC QLQ-C30 global health Status)

The longitudinal analysis of quality of life (QoL) revealed significant improvements in the PD-1/PD-L1 inhibitor group compared with the multikinase inhibitor group. At baseline, both groups had comparable QoL scores (sorafenib/lenvatinib: 53.1 ± 13.9 vs. PD-1/PD-L1: 53.5 ± 14.1, *p* = 0.78). By 6 months, the PD-1/PD-L1 group showed a clinically meaningful increase in the QoL scores (mean change: +12.4 ± 4.2 vs. +2.1 ± 3.8 in the multikinase inhibitor group; adjusted mean difference (AMD): +10.3, 95% CI: 7.2–13.4, *p* < 0.001). The multikinase inhibitor group exhibited minimal changes in QoL, with transient decreases at 3 months that were likely attributable to treatment-related toxicity (Table 2 and Supplementary Fig. S2).

### Secondary outcomes: correlation between treatment duration and adverse events

#### Treatment duration and reasons for discontinuation

The PD-1/PD-L1 inhibitor group had a significantly longer median treatment duration than the multikinase inhibitor group (9.5 months, 95% CI: 8.2–11.1 vs. 5.8 months, 95% CI: 4.6–7.0; *p* < 0.001). Disease progression was the primary reason for discontinuation in both groups, although its incidence was lower in the PD-1/PD-L1 group (52.0% vs. 68.4%, *p* = 0.003). Toxicity-related discontinuations occurred more frequently in patients treated with multikinase inhibitors (24.3% vs. 12.5%, *p* = 0.008), driven by intolerable hypertension and hand–foot syndrome. The Kaplan–Meier analysis revealed superior overall survival (OS) in the PD-1/PD-L1 group (median OS: 18.2 vs. 12.5 months; HR = 0.62; 95% CI: 0.45–0.85; *p* = 0.002) (Supplementary Table S3 and Supplementary Fig. S 4–5 ).

#### Associations between adverse events and psychological outcomes

**Fig. 2:**
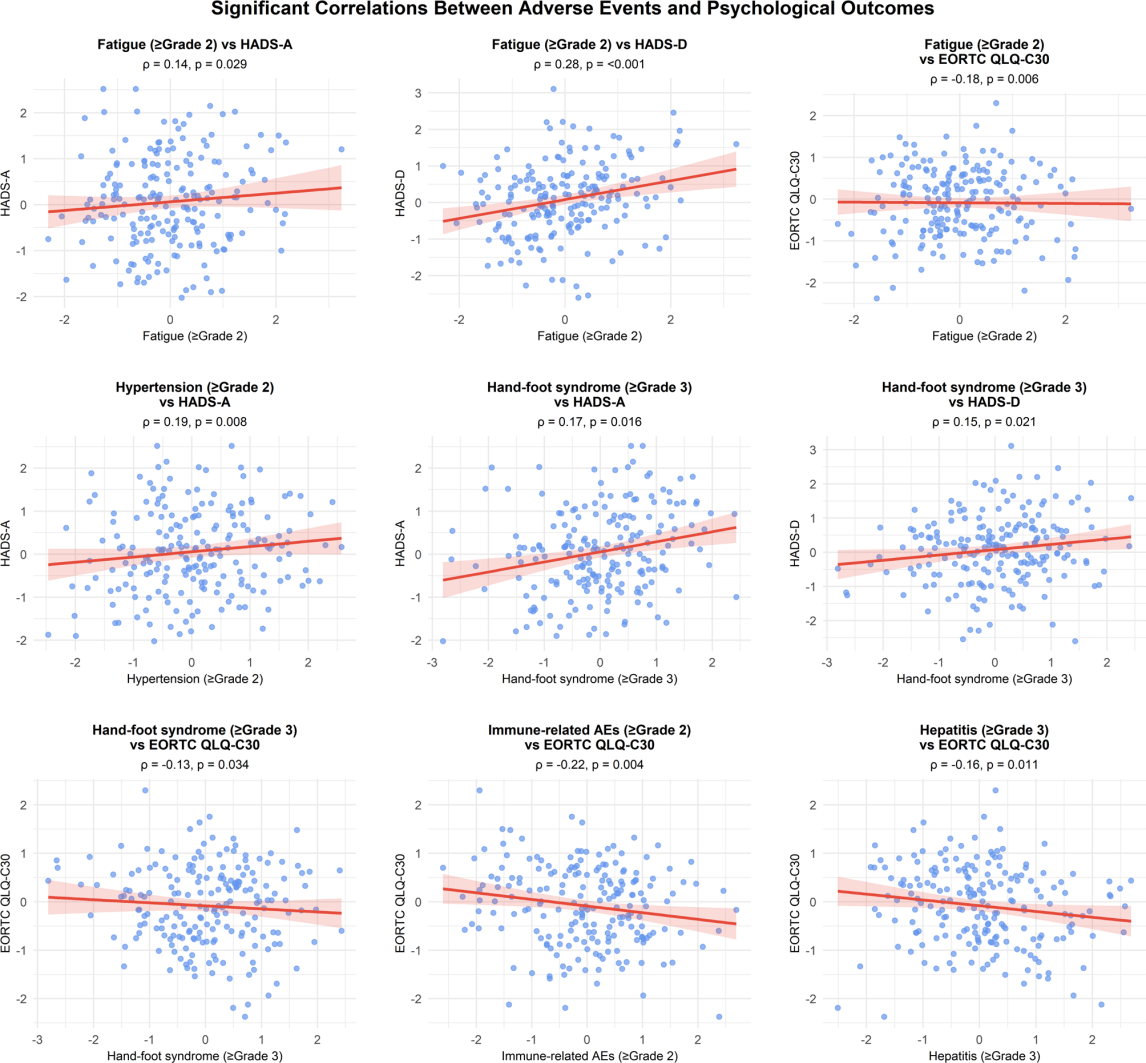
Correlation analysis between adverse events and psychological outcomes. Scatter plots show the relationships between adverse events (x-axis) and psychological measures (y-axis). Each panel displays the Spearman correlation coefficient (ρ) and the p value. HADS-A/D, Hospital Anxiety and Depression Scale–Anxiety/Depression subscales; EORTC QLQ-C30, European Organization for Research and Treatment of Cancer Quality of Life Questionnaire. The blue dots represent data points for individual patients; the red lines indicate linear regression trends. Significant correlations were observed between fatigue and depression (ρ = 0.28, *p* = 0.001), immune-related adverse events and quality of life (ρ=-0.22, *p* = 0.004), and hypertension/hand–foot syndrome with anxiety (*p* = 0.19, *p* = 0.008 and ρ = 0.17, *p* = 0.016, respectively).

Significant correlations were identified between specific adverse events (AEs) and psychological outcomes. Fatigue (CTCAE Grade ≥ 2) showed the strongest association with higher depression scores (HADS-D: Spearman’s ρ = 0.28, *p* < 0.001), whereas immune-related adverse events (irAEs) were inversely correlated with quality of life (EORTC QLQ-C30: ρ=−0.22, *p* = 0.004). Hypertension (Grade ≥ 2) and hand‒foot syndrome (Grade ≥ 3) were positively associated with anxiety scores (HADS-A: ρ = 0.19, *p* = 0.008 and ρ = 0.17, *p* = 0.016, respectively) (Table 3; Fig. 2).

#### Multivariate logistic regression analysis of fatigue and depression

The multivariate logistic regression analysis confirmed that fatigue (CTCAE Grade ≥ 2) independently predicted clinically significant depression (HADS-D score ≥ 8) after adjusting for age, the Child‒Pugh class, baseline tumour burden, and ECOG performance status. Patients with moderate-to-severe fatigue had 1.82-fold greater odds of developing clinically significant depression than those without fatigue (adjusted OR = 1.82, 95% CI: 1.24–2.68, *p* = 0.002). Correlations with other covariates, including Child‒Pugh class B/C (OR = 1.35, 95% CI: 0.88–2.07, *p* = 0.17) and bilobar tumour involvement (OR = 1.18, 95% CI: 0.79–1.76, *p* = 0.42), did not reach statistical significance (Table 4).

**Table 4:** Multivariate logistic regression analysis for clinically significant depression (HADS-D ≥ 8).

Variable	Adjusted OR	95% CI	*p*-value
Fatigue (Grade ≥ 2)	1.82	1.24–2.68	0.002
Age (per 5-year increase)	0.95	0.83–1.09	0.45
Child-Pugh class B/C	1.35	0.88–2.07	0.17
Tumor burden (Bilobar)	1.18	0.79–1.76	0.42
ECOG PS ≥ 2	1.52	0.97–2.38	0.067

Dependent variable: Clinically significant depression (HADS-D ≥ 8). Adjusted covariates: Age, Child-Pugh class, tumor burden, ECOG PS. OR: Odds ratio; CI: Confidence interval.

### Subgroup analysis stratified by treatment line (first-line vs. second-line therapy)

The subgroup analysis included adequate samples for first-line (*n* = 43 matched pairs) and second-line settings (*n* = 32 matched pairs) based on power calculations (a minimum of 30 pairs was needed to detect AMD ≥ 10 with 80% power, α = 0.05). The subgroup analysis stratified by treatment line revealed significant heterogeneity in the effect of PD-1/PD-L1 inhibitors on quality of life (QoL). Among patients receiving first-line therapy, those in the PD-1/PD-L1 group showed a marked improvement in QoL scores at 6 months compared with patients treated with multikinase inhibitors (adjusted mean difference [AMD]: +14.2, 95% CI: 10.5–17.9, *p* < 0.001). In contrast, for the patients receiving second-line therapy, the improvement in QoL was attenuated and nonsignificant (AMD: +3.8, 95% CI: −1.2 to 8.8, *p* = 0.13), with a significant treatment-by-line interaction (*p* < 0.001). Similar patterns were observed for anxiety/depression outcomes, where PD-1/PD-L1 therapy produced stronger psychological benefits when used in first-line settings (HADS-A interaction, *p* = 0.03; HADS-D interaction, *p* = 0.02) (Supplementary Table S4).

### Sensitivity analyses of missing data and model robustness

Sensitivity analyses comparing multiple imputation (MICE) and complete-case datasets produced consistent results across primary outcomes, supporting the robustness of our findings. For anxiety (HADS-A), the adjusted mean differences (AMDs) between treatment groups remained stable under both approaches: −2.5 (95% CI: −3.2 to − 1.8, *p* < 0.001) with multiple imputation versus − 2.4 (95% CI: −3.1 to − 1.7, *p* < 0.001) in the complete-case analysis. A similar concordance was observed for depression (HADS-D score: AMD = − 2.3 vs. −2.2, *p* < 0.001) and quality of life (QoL: AMD = + 10.3 vs. +10.1, *p* < 0.001). Less than 10% of the psychosocial data required imputation, and sensitivity models showed no substantial deviations in the effect estimates (Supplementary Table S5 and Supplementary Fig. S5).

## Discussion

This retrospective cohort study provides comprehensive evidence of the differential psychosocial and clinical effects of multikinase inhibitors (sorafenib/lenvatinib) and PD-1/PD-L1 inhibitors on patients with advanced HCC. Key findings indicate that compared with multikinase inhibitors, PD-1/PD-L1 inhibitors are associated with superior psychological outcomes, a prolonged treatment duration, and survival benefits, particularly when they are administered as first-line therapy. These results align with emerging insights into the interplay between immunotherapy, tumour microenvironment modulation, and systemic well-being^29^while also highlighting critical areas for clinical prioritization. The marked reductions in anxiety and depression scores (HADS-A/D scores) and improved QoL in the PD-1/PD-L1 group underscore the potential psychological advantages of immune checkpoint inhibitors. These findings contrast with those of prior studies that focused solely on survival outcomes, which often overlook psychosocial metrics^30^.

The observed improvement in QoL (AMD = + 10.3 at 6 months) exceeds the minimal clinically important difference (MCID = 8–10 points) for the EORTC QLQ-C30 Global Health Status scale^31^, suggesting that immunotherapy not only prolongs survival but also enhances patient-reported well-being in a clinically meaningful way. Similarly, the reduction in HADS-A scores (AMD=-2.4) meets the established MCID threshold of 1.5–2.5 points for the HADS instrument^32^, indicating that the improvement in anxiety experienced by patients receiving PD-1/PD-L1 inhibitors is likely to be perceptible and valuable to patients in their daily lives. These clinically significant improvements in patient-reported outcomes complement conventional efficacy metrics and provide a more comprehensive picture of treatment benefits beyond the tumour response and survival statistics. Importantly, these psychological benefits remained robust in sensitivity analyses using both multiple imputation and complete-case approaches, suggesting their validity, despite the limitations inherent to studies with retrospective designs.

Mechanistically, PD-1/PD-L1 inhibitors may mitigate tumour-driven systemic inflammation, which is implicated in depression and fatigue via cytokine-mediated pathways (e.g., IL-6 and TNF-α)^33^. This anti-inflammatory effect and durable tumour control likely contribute to sustained psychological benefits. The prolonged median treatment duration (9.5 vs. 5.8 months) and superior overall survival (OS) (18.2 vs. 12.5 months) of patients in the PD-1/PD-L1 group reinforce the efficacy of immunotherapy against advanced HCC. These results are consistent with those of phase III trials, such as IMbrave150 and CheckMate 459, which reported similar survival benefits with atezolizumab–bevacizumab and nivolumab, respectively^11,34^. However, our study uniquely links an extended treatment duration to reduced discontinuation due to progression (52.0% vs. 68.4%), emphasizing the role of immunotherapy in delaying therapeutic resistance. The lower toxicity-driven discontinuation rate (12.5% vs. 24.3%) further supports the safety profile of PD-1/PD-L1 inhibitors and is consistent with meta-analyses showing fewer high-grade adverse events associated with these inhibitors than those associated with tyrosine kinase inhibitors^35^.

The pronounced QoL and psychological benefits of first-line PD-1/PD-L1 inhibitors (AMD = + 14.2 for QoL) versus the attenuated effects of second-line therapy (AMD = + 3.8) suggest that early immunotherapy initiation maximizes psychosocial and clinical outcomes. This heterogeneity may reflect several interconnected factors. First, differences in disease control rates between treatment lines could contribute to the observed psychological outcomes, as a tumour response is often associated with better mental health in patients with advanced disease.

Second, the immunotherapy-naive status of patients receiving first-line treatment likely preserves antitumour T-cell functionality, whereas prior multikinase inhibitor exposure may induce immunosuppressive mechanisms (e.g., regulatory T-cell infiltration and VEGF-mediated exhaustion)^36,37^.

Third, the varying toxicity profiles between treatment settings may influence patient-reported outcomes, as treatment-related adverse events can significantly impact quality of life. While our subgroup analysis included adequate numbers of patients treated in first-line and second-line settings based on the power calculations, we acknowledge that larger cohorts would strengthen these findings. Nevertheless, the interaction p values underscore the importance of the treatment sequence, providing an additional patient-centred rationale for considering PD-1/PD-L1 inhibitors as the first-line standard of care, as endorsed by recent BCLC updates^23^.

The strong association between fatigue (Grade ≥ 2) and clinically significant depression (OR = 1.82) highlights fatigue as a modifiable risk factor for psychological morbidity. This result is consistent with studies linking cancer-related fatigue to dysregulated hypothalamic‒pituitary‒adrenal (HPA) axis activity and chronic inflammation^38^. Conversely, immune-related adverse events (irAEs) correlated with a reduced QoL, likely because of their management burden (e.g., corticosteroids for hepatitis/pneumonitis). However, irAEs may also signal robust immune activation, as evidenced by their association with the prolonged survival of patients with melanoma or NSCLC^39^, a paradox that warrants exploration in patients with HCC.

The efficacy of PD-1/PD-L1 inhibitors is intrinsically tied to the immune context of HCC, which may directly influence the psychosocial outcomes we observed. Our findings of significantly greater QoL improvements (AMD = + 14.2) and anxiety reduction (AMD=-3.7) with first-line immunotherapy are consistent with the immunobiological understanding of treatment sequencing. First-line PD-1/PD-L1 inhibitors are likely to result in a more favourable immune microenvironment with functional tumour-infiltrating lymphocytes (TILs), particularly CD8^+^ T cells, which are critical mediators of the therapeutic response^40^. The sustained psychosocial benefits we documented mirror the clinical response patterns typically associated with effective immune activation, where patients experience less symptom progression and a delayed deterioration of functioning^41^. This biological‒psychological connection is further supported by our observation of treatment line-dependent heterogeneity (interaction *p* < 0.001), which parallels recent single-cell studies showing that prior systemic therapies can reshape the immune landscape of HCC^42^. The attenuated psychological benefits in our second-line cohort (QoL AMD = + 3.8) may reflect a “cold” tumour microenvironment altered by previous treatments, particularly multikinase inhibitors that promote angiogenesis and stromal changes, limiting immune cell function^43^. These findings suggest that the psychological outcomes of HCC patients receiving immunotherapy may serve as patient-reported surrogates for underlying immunobiological processes, highlighting the importance of treatment sequencing not only for survival outcomes but also for maintaining psychological well-being. Future prospective studies that integrate both psychological metrics and immune biomarkers may further elucidate these connections and identify patients most likely to experience comprehensive benefits from PD-1/PD-L1 inhibition^44^.

This study has several limitations. First, its retrospective design introduces potential selection bias, despite the use of propensity score matching. While PSM helped balance the observable baseline characteristics, it has inherent limitations, including reduced statistical power due to the sample size reduction after matching and the inability to account for unmeasured confounders.

Treatment decisions were made by clinicians based on institutional practices, patient preferences, and resource availability, potentially introducing treatment selection bias that cannot be fully mitigated by statistical methods alone. Second, psychosocial assessments were conducted at fixed intervals, possibly missing transient fluctuations in anxiety or QoL.

Third, our study period (2018–2023) included significant changes in HCC treatment landscape. The publication of the IMbrave150 trial results in 2020 established atezolizumab+bevacizumab as the new first-line standard of care, which may have altered treatment selection patterns in the latter part of our study. These temporal shifts in treatment guidelines and drug availability likely influenced physician decision-making and patient expectations, potentially affecting both treatment allocation and psychological outcomes. For example, patients treated after the widespread adoption of immunotherapy combinations might have different expectations regarding efficacy and side effects than those treated earlier. Fourth, although our overall sample size was adequate for primary analyses, the statistical power for certain subgroup analyses (particularly treatment line-specific comparisons) may be limited, warranting a cautious interpretation of the findings on heterogeneity. Finally, regional variations in drug accessibility and reimbursement policies during the study period may have created disparities in treatment timing and selection that are not fully captured in our analysis, potentially limiting the generalizability of our findings to health care settings with different access patterns.

## Conclusions

In patients with advanced HCC, PD-1/PD-L1 inhibitors outperform multikinase inhibitors in reducing psychological distress, improving QoL, and prolonging survival, particularly when administered as first-line therapy. The interplay between immunotherapy, tumour microenvironment dynamics, and systemic inflammation provides a plausible biological basis for these benefits. Future studies should integrate psychosocial metrics with immunogenomic profiling to identify predictors of holistic patient outcomes. These findings support earlier immunotherapy adoption and the prioritized management of fatigue to mitigate the depression risk.

## Supplementary Information

Below is the link to the electronic supplementary material.

Supplementary Material 1

Supplementary Material 2

## Data Availability

All data generated or during this study are included in the manuscript or upplementary information. Further inquiries can be directed to corresponding author.
