# Supplementary material for: Psychological and quality of life outcomes associated with multikinase inhibitors versus immune checkpoint inhibitors in advanced hepatocellular carcinoma
Source: Sci Rep. 2026 Feb 12;16:8575. doi: 10.1038/s41598-026-39864-y (PMC12976292; doi:10.1038/s41598-026-39864-y)
Supplement: Supplementary file 1 — Supplementary Material 1 [file 41598_2026_39864_MOESM1_ESM.docx]

Supplementary Table 1: Linear Mixed-Effects Model Specifications and Diagnostics

| **Model Component** | **HADS-Anxiety** | **HADS-Depression** | **EORTC QLQ-C30 Global Health** |
| --- | --- | --- | --- |
| Fixed Effects | Treatment group, Time (months), Treatment×Time, Age, Child-Pugh class, Tumor burden | Treatment group, Time (months), Treatment×Time, Age, Child-Pugh class, Tumor burden | Treatment group, Time (months), Treatment×Time, Age, Child-Pugh class, Tumor burden |
| Random Effects | Random intercept and slope by patient ID | Random intercept and slope by patient ID | Random intercept and slope by patient ID |
| Covariance Structure | Unstructured | Unstructured | Unstructured |
| Model Diagnostics |  |  |  |
| Normality test (Shapiro-Wilk) | W=0.986, *p*=0.213 | W=0.991, *p*=0.345 | W=0.984, *p*=0.187 |
| Homoscedasticity (Levene’s test) | F=0.93, *p*=0.421 | F=1.02, *p*=0.392 | F=0.86, *p*=0.457 |
| Model Fit |  |  |  |
| AIC | 2431.2 | 2389.7 | 3215.8 |
| BIC | 2476.5 | 2434.9 | 3261.1 |
| Assessment Compliance |  |  |  |
| Baseline | 115/115 (100%) | 115/115 (100%) | 115/115 (100%) |
| 3-month | 108/112 (96.4%) | 108/112 (96.4%) | 107/112 (95.5%) |
| 6-month | 104/109 (95.4%) | 104/109 (95.4%) | 103/109 (94.5%) |
| 9-month | 98/103 (95.1%) | 97/103 (94.2%) | 97/103 (94.2%) |
| 12-month | 91/96 (94.8%) | 90/96 (93.8%) | 89/96 (92.7%) |
| Missing Data Pattern | MCAR test: χ²=8.32, *p*=0.403 | MCAR test: χ²=7.69, *p*=0.465 | MCAR test: χ²=8.97, *p*=0.345 |

Note: MCAR = Missing Completely At Random, tested using Little’s MCAR test. Compliance rates represent completed assessments/scheduled assessments. Model diagnostics indicate adequate fit with residuals meeting normality and homoscedasticity assumptions.

Supplementary Table 2: Longitudinal changes in HADS anxiety and depression scores

| **Timepoint** | **HADS-A Score** | | | | **HADS-D Score** | | | |
| --- | --- | --- | --- | --- | --- | --- | --- | --- |
|  | Sorafenib/Lenvatinib (n=152) | PD-1/ PD-L1 (n=152) | AMD  (95% CI) | *p*- value | Sorafenib/Lenvatinib (n=152) | PD-1/ PD-L1 (n=152) | AMD  (95% CI) | *p* - value |
| **Baseline** | 8.5 ± 2.3 | 8.4 ± 2.1 | – | – | 7.8 ± 2.1 | 7.7 ± 2.3 | – | – |
| **3 months** | 8.2 ± 2.4 | 6.9 ± 2.0 | −1.3 (−1.9 to −0.7) | <0.001 | 7.6 ± 2.2 | 6.4 ± 1.9 | −1.2 (−1.8 to −0.6) | <0.001 |
| **6 months** | 8.0 ± 2.5 | 5.2 ± 1.8 | −2.4 (−3.1 to −1.7) | <0.001 | 7.5 ± 2.3 | 5.1 ± 1.7 | −2.3 (−3.0 to −1.6) | <0.001 |
| **Discontinua- tion** | 9.1 ± 2.7 | 6.0 ± 2.2 | −2.1 (−2.8 to −1.4) | <0.001 | 8.3 ± 2.5 | 5.5 ± 1.9 | −2.2 (−2.9 to −1.5) | <0.001 |

Notes: Data presented as mean ± SD. AMD = Adjusted mean difference (PD-1/PD-L1 vs. sorafenib/lenvatinib) derived from linear mixed-effects models, adjusted for age, Child-Pugh class, and baseline tumor burden. HADS-A = Hospital Anxiety and Depression Scale-Anxiety subscale; HADS-D = Depression subscale. *p*-values for intergroup comparisons at each timepoint.

Supplementary Table 3:　Treatment Duration, Discontinuation Reasons, and Survival Outcomes

| **Parameter** | **Sorafenib/Lenvatinib (n=152)** | **PD-1/PD-L1 (n=152)** | ***p*-value** |
| --- | --- | --- | --- |
| **Median treatment duration (months)** | 5.8 (4.6–7.0) | 9.5 (8.2–11.1) | <0.001 |
| **Discontinuation reasons, n (%)** |  |  |  |
| - Disease progression | 104 (68.4%) | 79 (52.0%) | 0.003 |
| - Toxicity | 37 (24.3%) | 19 (12.5%) | 0.008 |
| - Death | 8 (5.3%) | 5 (3.3%) | 0.38 |
| - Other | 3 (2.0%) | 4 (2.6%) | 0.70 |
| **Median OS (months)** | 12.5 (10.8–14.3) | 18.2 (15.9–20.5) | 0.002 |
| **HR for death (95% CI)** | Reference | 0.62 (0.45–0.85) | 0.002 |

Notes: Data presented as median (95% CI) or n (%). OS = Overall survival; HR = Hazard ratio derived from Cox proportional hazards model adjusted for age and Child-Pugh class. Treatment duration compared via log-rank test; discontinuation reasons analyzed via chi-square test. *p*<0.05 considered statistically significant.

**Supplementary Table 4.** Subgroup Analysis of Primary Outcomes by Treatment Line

| **Outcome** | **First-line Therapy** | **Second-line Therapy** | **Interaction p-value** |
| --- | --- | --- | --- |
| **EORTC QLQ-C30 (QoL)** | AMD: +14.2 (10.5–17.9), p<0.001 | AMD: +3.8 (−1.2–8.8), p=0.13 | <0.001 |
| **HADS-A (Anxiety)** | AMD: −3.5 (−4.2–−2.8), p<0.001 | AMD: −1.1 (−2.0–−0.2), p=0.02 | 0.03 |
| **HADS-D (Depression)** | AMD: −3.2 (−3.9–−2.5), p<0.001 | AMD: −0.9 (−1.8–0.0), p=0.06 | 0.02 |

Notes: AMD: Adjusted mean difference (PD-1/PD-L1 vs. sorafenib/lenvatinib) derived from linear mixed-effects models, adjusted for age, Child-Pugh class, and baseline tumor burden. Interaction p-values test whether treatment effects differ between first-line and second-line subgroups.

**Supplementary Table 5.** Sensitivity analysis comparing the results obtained from multiple imputation and complete-case analyses

| **Outcome** | **Multiple Imputation (MICE)** | **Complete-Case Analysis** | **Difference (ΔAMD)** |
| --- | --- | --- | --- |
| **HADS-A (Anxiety)** | −2.5 (−3.2 to −1.8), p<0.001 | −2.4 (−3.1 to −1.7), p<0.001 | −0.1 (−0.3 to 0.1) |
| **HADS-D (Depression)** | −2.3 (−3.0 to −1.6), p<0.001 | −2.2 (−2.9 to −1.5), p<0.001 | −0.1 (−0.4 to 0.2) |
| **EORTC QLQ-C30 (QoL)** | +10.3 (7.2–13.4), p<0.001 | +10.1 (6.9–13.3), p<0.001 | +0.2 (−0.5 to 0.9) |

Notes: AMD: Adjusted mean difference (PD-1/PD-L1 vs. sorafenib/lenvatinib). Difference (ΔAMD): Multiple imputation AMD minus complete-case AMD (with 95% CI). Missing data: 8.7% of HADS assessments and 9.2% of QoL assessments required imputation.
