# Supplementary material for: Psychological and quality of life outcomes associated with multikinase inhibitors versus immune checkpoint inhibitors in advanced hepatocellular carcinoma
Source: Sci Rep. 2026 Feb 12;16:8575. doi: 10.1038/s41598-026-39864-y (PMC12976292; doi:10.1038/s41598-026-39864-y)

**Supplementary Fig. S1.** Forest plot of adjusted odds ratios (ORs) for clinically significant anxiety and depression (HADS ≥ 8) comparing PD-1/PD-L1 inhibitors with sorafenib/lenvatinib across time points. Models were adjusted for age, Child–Pugh class, tumor burden, and ECOG performance status. ORs < 1 indicate lower psychological risk with PD-1/PD-L1 therapy. Error bars show 95% confidence intervals, and the dashed line denotes no between-group difference (OR = 1.0).

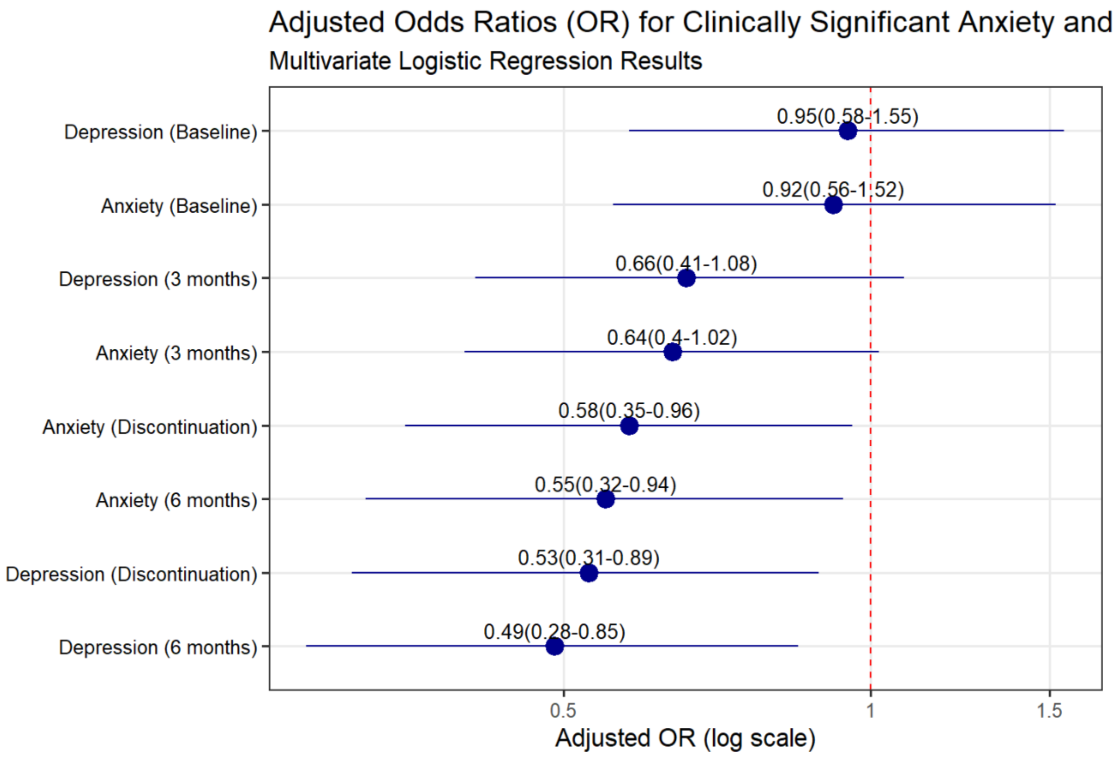

**Supplementary Fig. S2.** Longitudinal changes in the EORTC QLQ-C30 Global Health Status scores of patients treated with PD-1/PD-L1 inhibitors compared with those treated with sorafenib/lenvatinib. The data are presented as the means ± SDs. Higher scores indicate better quality of life (range 0–100). The statistical analysis was performed using linear mixed-effects models adjusted for the baseline characteristics.

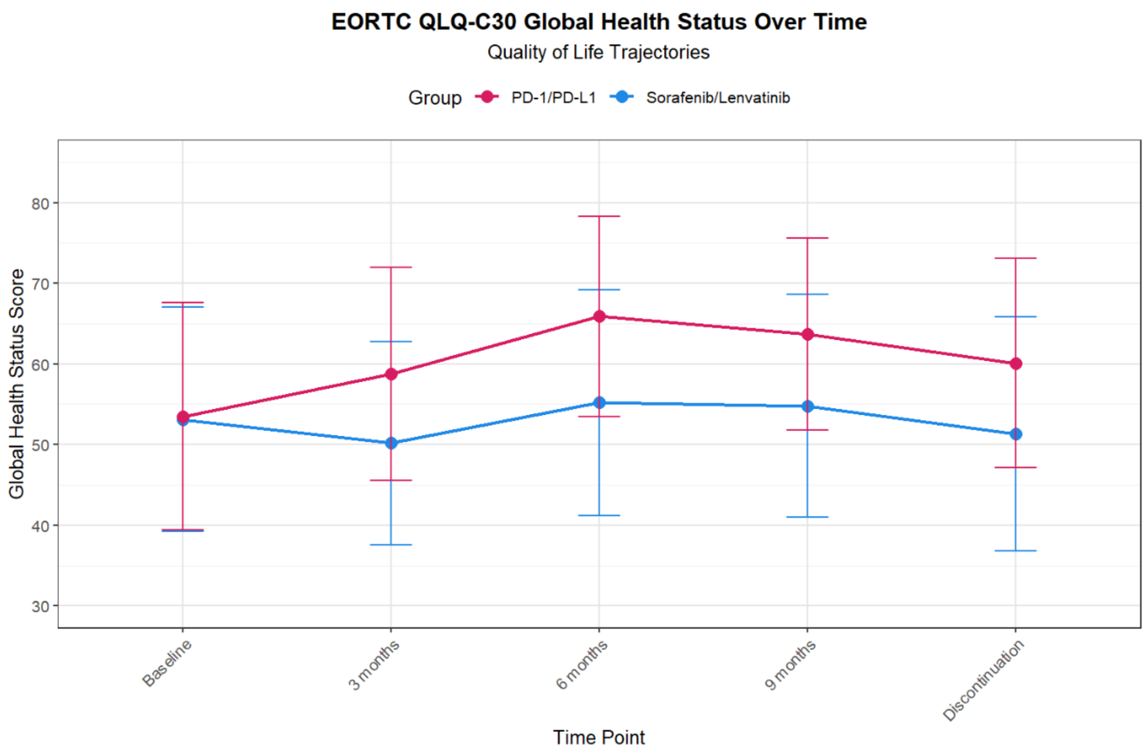

**Supplementary Fig. S3.** Reasons for treatment discontinuation in patients receiving PD-1/PD-L1 inhibitors versus sorafenib/lenvatinib. The bar graph shows the percentage of patients in each group who discontinued treatment for different reasons. The statistical significance of differences between groups is indicated as **P < 0.01 and ns = not significant.

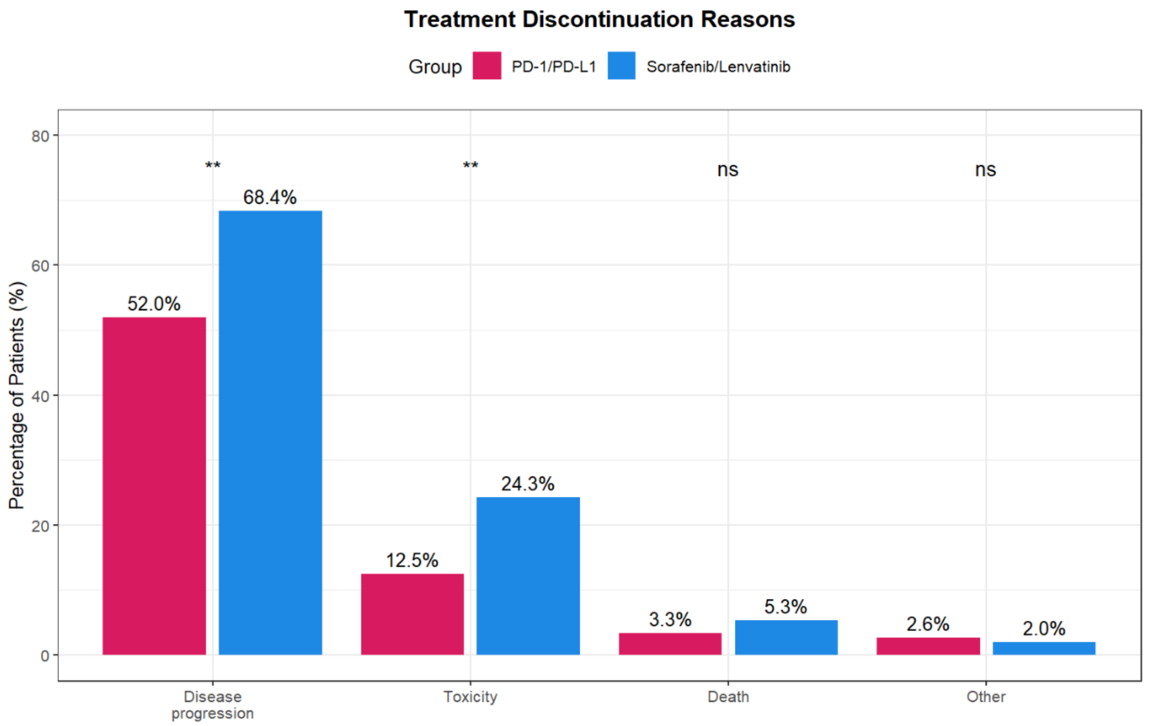

**Supplementary Fig. S4.** Comparison of overall survival and the treatment duration between the PD-1/PD-L1 inhibitor and sorafenib/lenvatinib groups. Box plots show median values with 95% confidence intervals. The left panel shows the overall survival in months; the right panel shows the treatment duration in months. The values above the boxes indicate the medians (95% CIs). The statistical significance of differences between groups is indicated as **P < 0.01 and ***P < 0.001.

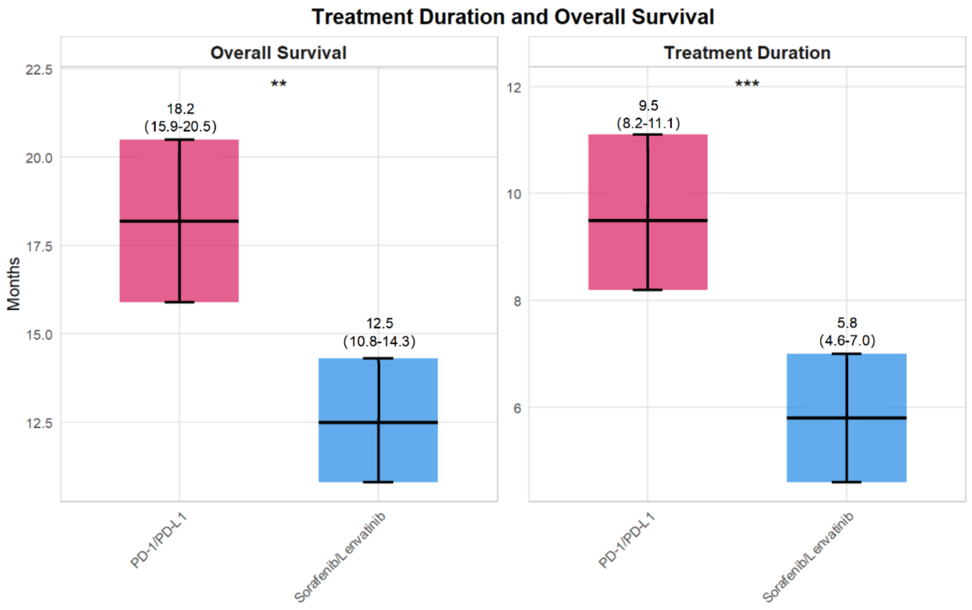

**Supplementary Fig. S5.** Sensitivity analysis of primary psychological outcomes comparing PD-1/PD-L1 inhibitors with sorafenib/lenvatinib using multiple imputation and complete-case analyses. The forest plot shows adjusted mean differences (AMDs) with 95% confidence intervals. Negative HADS values and positive EORTC QLQ-C30 values indicate symptom improvement and better quality of life, respectively. Consistent estimates across methods (<10% difference) demonstrate the robustness of the findings.

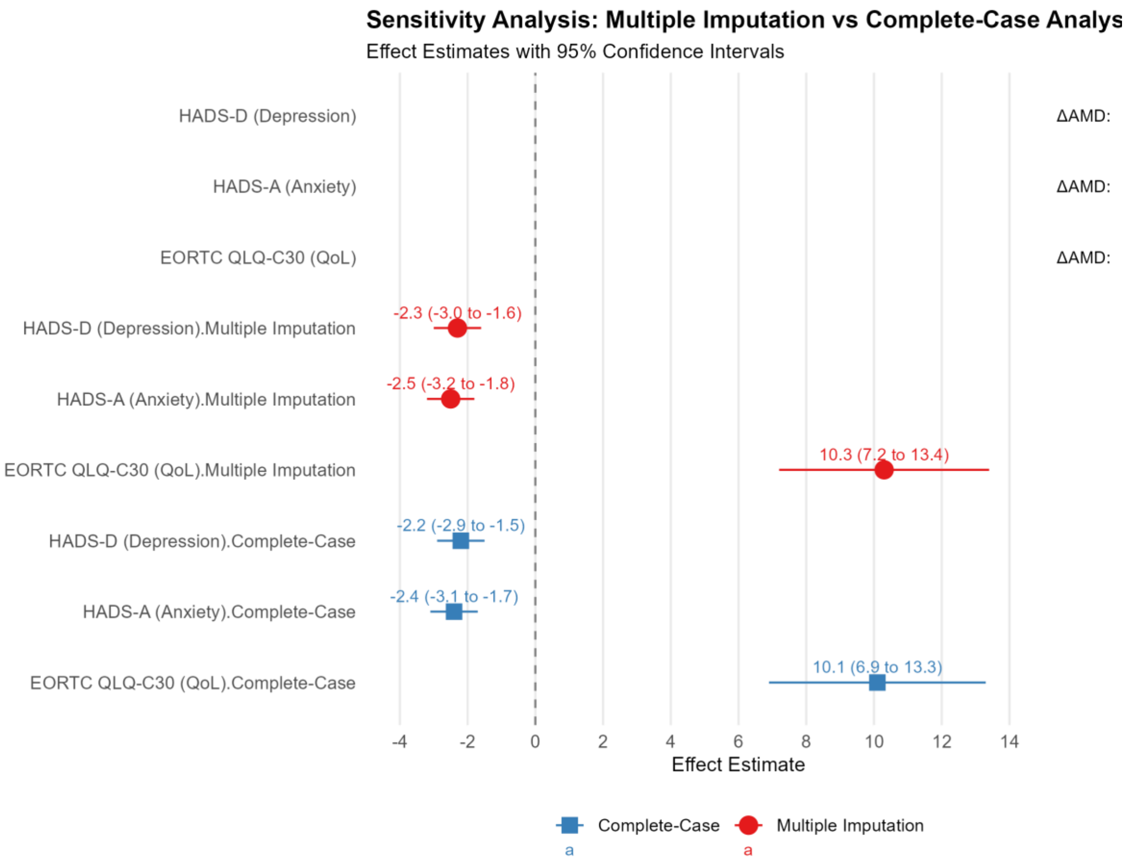

Supplement: Supplementary file 2 — Supplementary Material 2 [file 41598_2026_39864_MOESM2_ESM.pdf]
